# The Housing of Staffs in Mental Hospitals

**Published:** 1913-02-01

**Authors:** 


### The Hospital

The Workers' Newspaper of Administrative Medicine and Institutional Life, Administration, National Insurance and Health.

No. 1387, Vol. LIII. SATURDAY, FEBRUARY 1, 1913. PRICE ONE PENNY.

### THE HOUSING OF STAFFS IN MENTAL HOSPITALS.

The care of the mentally afflicted is an occupation always responsible, often arduous, sometimes even dangerous. It is essential that those who follow it should have such an amount of time free from duty as will enable them to shake off the effects of their work and allow fair opportunities for recuperation. If this be not done, both attendant, nurse, and patient will inevitably suffer. No attendant can work properly when wearied and overstrung. Irritability is the certain result. Of all forms that of mental nursing is the least compatible with such a condition. It should not be necessary for the staff to be compelled to go afield when not actually at work in order to escape from the atmosphere and influence of the mental hospital. There are days when the weather forbids leaving the institution; days when one is disinclined to go out or has some indoor occupation. Those who follow this profession are doing such a public service as may fairly entitle them to claim that the conditions under which they work shall be the best obtainable.

Is the accommodation provided for attendants and nurses in mental hospitals generally such as may reasonably be claimed? There are instances where suitable accommodation has been provided with obvious benefit to the institution which has insisted upon it. At one time all mental workers lived in close contact and even association with the patients. By day the influence of the depressing surroundings was never absent, and by night their rest was liable to be broken by the cries and noise of the patients. This state of things cannot be entirely done away with. Some at least of the staff must occupy rooms close to the patients in order to afford assistance to those on night duty in emergencies. These need not, however, always be the same. It is clearly understood by those affected that the nature of their calling necessitates that a portion at least of the staff of a mental hospital must sleep within call of the night nurses. If, fully recognising this fact, the mental workers generally were to demand that such arrangements should be made as would obviate this necessity being imposed upon all, could such a claim be described as unreasonable? It must be granted that such a request would be a reasonable one. It must also be admitted that it is one which no public authority, however careful it might be in its expenditure of public money, could deny. If the matter were accurately understood there would be no desire to refuse.

A contented and healthy staff is an immense asset in the working of a mental hospital. Its money value is difficult to estimate, and the fulfilment of such a requirement for the staff would certainly operate in the attraction of a better-class nurse. Expenditure may take the form of a capital sum spent on buildings, or it may be given as payments to the staff in lieu of lodging. The authority may provide a home away from the wards, or may pay the staff such a sum as will enable them to make adequate arrangements for living outside the precincts of the mental hospital. The advantages of this principle are already recognised. Cottages are provided for the married attendants, or they are paid lodging-money when no cottages are vacant. It means that the best men are thus retained in the service of the institution. The system works so well as to suggest the advisability of extending it. Many utter, however, strong objections to its applicability to the staff generally, and more especially to the junior staff of both sexes. With the exception of those who have parents or relatives with whom they could lodge near by, this system could not be applied to the younger members of the female staff, and would be inadvisable for some who had passed that stage. In institutions where young women are taken in to train, many of whom have left home for the first time, there are grave objections to it. Every administrator recognises the advantage of allowing his staff as much freedom as possible when the day's duty is done.

Many administrators have longed for some rational means of interesting their junior female staffs when off duty, and especially in the dark winter evenings. But the above objections would only be increased by a system which entailed greater liberty for those unfitted for it, together with less supervision. It would not be one which would induce mothers to send their daughters for training to an institution where it was in vogue. There are also frequent objections to its general application to the junior members of the male staff. It is a method which under suitable circumstances might well be employed to reward tried and trustworthy nurses of both sexes. It might easily and with advantage be used for all members of the night staff. It is not, however, capable of such general application as to obviate the necessity of doing something more. The best alternative remedy is the provision of separate blocks for male and female nurses, at such a distance from the wards as will effectually cut them off from the hospital atmosphere when off duty. In such buildings attendants can be afforded comfortable surroundings in a way which is impossible when they are scattered about the wards of a large building. They can there enjoy all reasonable freedom in their leisure hours and yet be under that supervision which is necessary. Such separate blocks have been found useful in general hospitals; there is a greater necessity for them in mental hospitals. Whatever remedy is provided it is certain that money will be spent. It is equally certain that it will be money well spent.

